# Vacuum‐Assisted Excision and Assessing Residual Tumor Burden in Patients With Breast Cancer Following Neoadjuvant Chemotherapy (A Pilot Study)

**DOI:** 10.1155/ijbc/9951029

**Published:** 2026-04-25

**Authors:** Hawzhin Hashemi, Asiie Olfatbakhsh, Shiva Moghadam, Alireza Aghanajafi, Laila Heydari, Marzieh Rostami, Shahpar Haghighat

**Affiliations:** ^1^ Breast Disease Department, Breast Cancer Research Center, Motamed Cancer Institute, ACECR, Tehran, Iran, acecr.ac.ir; ^2^ Quality of Life Department, Breast Cancer Research Center, Motamed Cancer Institute, ACECR, Tehran, Iran, acecr.ac.ir

## Abstract

**Aim:**

The objective of this study is to evaluate the accuracy of vacuum‐assisted excision as a minimally invasive method for assessing residual tumor burden in distinct breast cancer subtypes following neoadjuvant chemotherapy.

**Materials and Methods:**

In this pilot clinical trial, 20 patients with breast cancer scheduled for neoadjuvant chemotherapy were assessed. Upon completion of chemotherapy, patients underwent ultrasound‐guided vacuum‐assisted excision of the tumor site, performed by a radiologist. Subsequently, surgical excision of the tumor was carried out. The pathology reports from the vacuum excision were compared with the surgical specimens to determine the concordance in detecting residual tumor tissue.

**Results:**

Among the 20 patients who underwent vacuum‐assisted excision, 13 patients demonstrated no residual tumor in both vacuum pathology and surgical pathology. However, in four patients, including three cases of Ductal Carcinoma In Situ (DCIS) and one case of Invasive Ductal Carcinoma (IDC), a false negative vacuum excision was reported. In three patients, residual tumor was reported both in surgical and vacuum pathology. The positive predictive value, negative predictive value, and accuracy of vacuum excision for detecting residual tumor were 100%, 76.5%, and 80%, respectively. The sensitivity and specificity of vacuum excision were 42.9% and 100%, respectively.

**Conclusion:**

Based on the findings of this study and considering the accuracy of vacuum excision in identifying residual tumors (80%), it is evident that vacuum excision cannot currently serve as a substitute modality for surgery in the management of patients with post‐neoadjuvant breast cancer. Further research with a larger sample size is warranted to enhance our understanding in this area.

**Trial Registration:**

IRCT20241204063942N1

## 1. Introduction

Breast cancer stands as the most prevalent cancer and the fifth leading cause of mortality among Iranian women, with approximately 25% succumbing to the disease despite advancements in diagnosis and treatment [1]. Notably, breast cancer emerges as the most fatal cancer in women aged 40–55, contributing to the loss of 1200 lives annually in Iran [2–4]. The current treatment landscape for breast cancer encompasses a multifaceted approach including surgery, chemotherapy, radiotherapy, and hormone therapy, with the sequence and necessity of these modalities tailored to the disease stage, pathology type, patient age, and physical well‐being. Typically, early‐stage patients without axillary lymph node involvement or distant metastases undergo primary surgery followed by adjunctive therapies. Conversely, individuals presenting with advanced disease characterized by a substantial tumor burden or extensive axillary lymph node involvement may first undergo neoadjuvant chemotherapy (NAC) to facilitate tumor and lymph node size reduction.

In recent years, the utilization of NAC has increasingly been used in cancer patient management. Its efficacy in tumor size reduction has been unequivocally demonstrated, particularly in individuals with locally advanced breast cancer or those ineligible for breast‐conserving surgery due to tumor size constraints. The administration of NAC in these scenarios not only influences tumor response but also impacts the overall survival outcomes for patients undergoing surgical intervention [5, 6].

The tumor response rate to a chemotherapy regimen holds significant prognostic value in tailoring individualized treatment strategies and averting the utilization of ineffective therapies in patients [3–5]. Specifically, the ability to predict the tumor response aids in optimizing treatment selection and minimizing the administration of futile interventions. Notably, patients demonstrating a complete pathological response (PCR), following NAC are anticipated to achieve superior treatment outcomes compared with non‐responders. Achieving complete response to treatment is a key sign that the treatment is working well and leads to better patient outcomes [6]. Therefore, accurately measuring the remaining tumor after chemotherapy helps surgeons decide how much tissue to remove during surgery. This information guides surgeons to perform more conservative resections, thereby minimizing unnecessary tissue excision, achieving clear surgical margins, and mitigating undue patient morbidity.

Moreover, over the past decade, the implementation of NAC in specific subsets of patients with breast cancer has resulted in the complete eradication of tumors in pathology. Studies have highlighted PCR rates exceeding 60% in Triple Negative (TN) and HER2+ breast cancer subgroups, prompting deliberation on the essentiality of surgical intervention following NAC in these particular patient cohorts [7–9].

In the clinical decision‐making process for these patients, local recurrence must be carefully considered. Previous studies examining this issue have encountered methodological challenges, notably the absence of rigorous patient selection criteria and overreliance on physical examination alone for residual tumor assessment, which has inherent limitations. Additionally, the deficiency in utilizing imaging‐guided sampling techniques has impeded accurate tumor evaluation. The primary obstacle in establishing definitive surgical strategies in recent years has been the absence of a standardized imaging modality for the precise determination of residual tumor burden [4, 10–12].

Currently, the gold standard for assessing residual tumor burden post‐NAC remains histopathological analysis [13]. While various studies have explored the utility of imaging modalities in evaluating NAC response, a consensus on the most accurate imaging technique is yet to be established. The concept of early PCR remains a topic of debate within the scientific community [3, 4].

During the preoperative evaluation of patients, a variety of imaging modalities, including mammography, ultrasound, and magnetic resonance imaging (MRI), are employed. MRI stands out as a preferred imaging modality for accurately determining tumor size, showing good correlation with pathology reports. It is widely endorsed as a reliable method for this purpose [14, 15]. However, MRI’s ability to distinguish tumoral from fibrotic tissue resulting from scarring is limited [10, 16]. Consequently, while imaging methods offer valuable insights, they may not definitively ascertain the presence of residual viable tumor following NAC, underscoring the continued necessity for histopathological examination as the most accurate diagnostic tool.

Over the past two decades, the integration of core needle biopsy (CNB) guided by imaging modalities such as ultrasonography, mammography, and MRI has revolutionized the preoperative evaluation of benign and malignant breast lesions, providing surgeons with detailed diagnostic insights. In instances where vacuum‐assisted sampling is utilized (potentially yielding substantial tissue samples), and subsequent histopathological assessment reveals the complete removal of a benign lesion, the need for surgical intervention may be eliminated. Recent research has underscored the utility of vacuum sampling in evaluating residual viable tumor post‐chemotherapy, showcasing its potential in clinical decision‐making. Patients with breast cancer are stratified into distinct subgroups based on pathological features and histological markers, namely Luminal A, Luminal B, HER2+, and Triple‐Negative. Notably, HER2+ and Triple‐Negative subgroups exhibit greater responsiveness to NAC and higher rates of pathological complete response (PCR) than other subgroups. Across various investigations, the PCR rates in these subgroups have been documented to range between 30% and 60% [14, 17].

The conventional approach to establishing PCR currently involves surgical intervention post‐chemotherapy, serving both diagnostic and therapeutic purposes. Given the notable incidence of PCR in specific patient cohorts, verifying the absence of residual viable tumor in the tumor site prior to NAC can aid in determining the necessity of surgical excision. In recent years, emerging evidence has highlighted the utility of ultrasound or mammography‐guided sampling techniques, particularly utilizing the vacuum‐assisted method, with a diagnostic accuracy of approximately 97.5%, in confirming the presence of residual tumor tissue in the breast following chemotherapy [15].

Given the limitations of current imaging modalities in confirming the presence of viable tumor cells at the post‐chemotherapy tumor site and the reported diagnostic accuracy of MRI in predicting PCR is at only 74% in studies, the rationale for employing Vacuum Core Needle Biopsy (VCNB) to verify PCR appears justified [4, 10–12].

To validate PCR preoperatively, acquiring sufficient tissue from the primary tumor site, which is marked before NAC sampled via vacuum‐assisted excision (VAE) is essential.

Confirmation of PCR indicates eradication of viable tumor cells at the previous tumor site. Similarly, pre‐NAC CNB of axillary lymph nodes for initial assessment of lymph node involvement, followed by repeat CNB post‐NAC, offers insight into treatment response. Comparative analysis between vacuum excision pathology findings and surgical pathology results serves to validate the accuracy of this method in identifying residual viable tumor post‐chemotherapy.

This research study compared the pathological report of VAE in TN and HER2+ patient cohorts against post‐surgery pathology reports to determine residual tumor presence following NAC. The study findings hold implications for breast cancer subgroups exhibiting heightened NAC responsiveness (TN and HER2+), wherein PCR assessment aids in pre‐surgical decision‐making, potentially reevaluating the necessity of breast surgery in these specific breast cancer subtypes.

## 2. Materials and Methods

A pilot clinical trial study was conducted from June 2023 to January 2024, which involved patients who consented to NAC at the Motamed Cancer Institute, a leading center for cancer treatment and research in Iran. Eligible patients were women with a diagnosis of triple‐negative and/or HER2‐positive invasive ductal breast cancer, of any tumor size, with or without axillary lymph node involvement and without metastases, who were candidates for NAC. Exclusion criteria were as follows: multifocal, multicentric, and bilateral breast cancers and inflammatory carcinoma.

The study focused on individuals diagnosed with triple‐negative and HER2+ breast cancer subtypes. Following marker placement and completion of NAC, clinical and ultrasonographic assessments confirmed the absence of residual tumor. Vacuum sampling procedures were conducted by a specialized radiologist under ultrasound guidance using a Core Needle. Tissue surrounding the pre‐chemotherapy marker site in the breast was vacuumed and sent for pathological analysis. Subsequently, patients underwent surgical procedures as part of their treatment plan.

The outcome of interest was the pathology report in two techniques of sampling.

The sample size of 20 patients for the current study was determined based on similar previous research. According to the Kuerer HM et al. study, in 95% of cases, vacuum sampling is consistent with surgical sampling, and both methods confirm complete patient recovery. Considering an alpha coefficient of 5% and an error rate of 10%, the necessary sample size was calculated to be 20 participants [15].

The data for the current investigation were extracted from patients’ electronic medical records. A comparative analysis was conducted to assess pathology results obtained by vacuum sampling and by standard pathology methods.

This study was approved by the ethics committee of Motamed Cancer Institute and received the institutional review board code of IR.ACECR.IBCRC.REC.1399.011. Before enrollment, patients were thoroughly informed about the study’s purpose and procedures. Those who agreed to partake signed an informed consent form. Patients were assured that their participation would not incur any additional costs, as vacuum excision would be provided at no charge. Additionally, we discussed potential side effects of the procedure, such as bleeding, bruising, and post‐excision pain, to ensure patients were aware of possible discomfort associated with the method. All patients who entered the study signed an informed consent form.

Radiologic Complete Response is considered based on the radiologist’s opinion by performing an ultrasound and the absence of a tumor of a certain size at the location of the marker embedded in the tumor. Vacuum excision was performed by radiologist under ultrasound guidance and local anesthesia.

If the ultrasound shows that the tumor has not been completely eliminated by chemotherapy and there is a partial response, the patient is excluded from the study and vacuum excision will not performed.

### 2.1. Statistical Analysis

Descriptive statistics were employed to report the absolute and relative frequencies of complete PCRs achieved through the two sampling techniques. To evaluate the relationship between the frequencies of complete responses, Fisher’s test was used to determine statistical significance, with *p* < 0.05 considered significant. The accuracy indices of vacuum excision were calculated using surgical pathology as the gold standard.

## 3. Results

A total of 20 patients, comprising 10 TN and 10 patients with HER2+ breast cancer, underwent vacuum excision procedures, post‐neoadjuvant chemotherapy under ultrasound guidance from the breast marked site. The mean age of the patients was 34.8 years (±4.8).

The clinical characteristics of the patients are detailed in Table 1.

**Table 1:** Patient clinical characteristics.

Variable	Number	Percent
Age (year)		
30–50	11	55
≥ 50	9	45
Tumor size (cm)		
≤ 2	1	5
2–3	6	30
3–5	10	50
≥ 5	3	15
Pathology subgroup		
TN	10	50
HER2+	10	50
Grade		
1	1	5
2	16	80
3	3	15
Pathology type		
IDC	17	85
IDC + DCIS	3	15
Chemotherapy regimen		
(TN) AC∗4‐TC∗4	10	50
(HER2+) TC‐HP	10	50
Number of chemotherapy course		
6	9	45
8	11	55

Abbreviations: DCIS: ductal carcinoma in situ; HER2+: human epidermal growth factor receptor 2 positive; IDC: invasive ductal carcinoma; AC∗4‐TC∗4: Adriamycin–Cyclophosphamide‐Taxan–Carboplatin; TC‐HP; Taxoter‐Carboplatin‐Herceptin‐Perjeta; TN: triple negative.

The chi‐square test was employed for descriptive analysis, and the Fisher test was utilized for the analytical study.

Table 2 presents the frequency distribution of pathology types observed in vacuum excision and in surgical pathology. Notably, in cases involving invasive ductal carcinoma (IDC) and ductal carcinoma in situ (DCIS), a 100% concordance was noted where residual tumor absence was consistently reported in vacuum biopsies (*p* value = 0.004).

**Table 2:** Frequency of pathology type in surgical and vacuum biopsies.

Vacuum pathology	Surgical pathology			
	No residue	IDC	IDC + DCIS	Total (%)
No residue	13	1	3	17 (85)
IDC	0	3	0	3 (15)
Total	13	4	3	20 (100)
*p* value	0.004			

Abbreviations: DCIS: ductal carcinoma in situ; IDC: invasive ductal carcinoma.

Based on our findings, 20% of patients (four individuals) exhibited discordance between vacuum excision results and surgical pathology, with residual tumor identified solely in the latter. Among these cases, two patients presented with a combination of IDC and DCIS as their primary pathology, with subsequent surgical pathology indicating DCIS only, potentially reflecting limited chemotherapeutic efficacy of vacuum biopsies in this particular pathology subtype. Notably, two patients were classified as TN, while the remaining two were classified as HER2+. Furthermore, among the three patients with residual tumor, the primary tumor size exceeded 3 cm.

It is worth noting that in patients who have invasive and in situ carcinoma, the likelihood of the tumor remaining after vacuum excision is higher due to the presence of microcalcifications and the extent of the in situ component. At the same time, because of the limitations of the vacuum excision method in the complete excision of larger tumors, the possibility of residual tumor and false‐negative results increases.

Conversely, a significant concordance rate of 80% (16 patients), 13 patients with no tumor residue and three patients with tumor residue, was observed between vacuum excision pathology and surgical pathology reports (*p* value = 0.004), and discordance rate of 20% (four patients), was observed between vacuum excision pathology and surgical pathology reports.

According to data presented in Table 3, the sensitivity of vacuum excision is reported at 42.9%, meaning that in 57% of cases, the procedure fails to identify residual tumor presence. Conversely, the specificity of vacuum excision is recorded at 100%, signifying its ability to accurately confirm the absence of residual tumors in cases devoid of such pathology.

**Table 3:** Accuracy of vacuum excision in determining tumor residual.

Index	Mean percent	95% CI
Sensitivity	42.86	59.81–90.90
Specificity	100	100–29.75
Prevalence	35	22.59–39.15
Positive predictive value	100	100–24.29
Negative predictive value	76.47	19.93–10.50
Accuracy	80	27.94–34.56

The positive predictive value (PPV) of vacuum excision stands at 100%, implying that any indication of residual tumor in the excision results unequivocally corresponds to the presence of residual tumor. On the other hand, the negative predictive value (NPV) of vacuum excision is calculated at 76.5%, suggesting that if the vacuum excision results are negative for 100 patients, approximately 77 individuals are correctly classified as tumor‐free, while 23 individuals harbor residual tumor that remained undetected by the vacuum excision.

**Figure 1:**
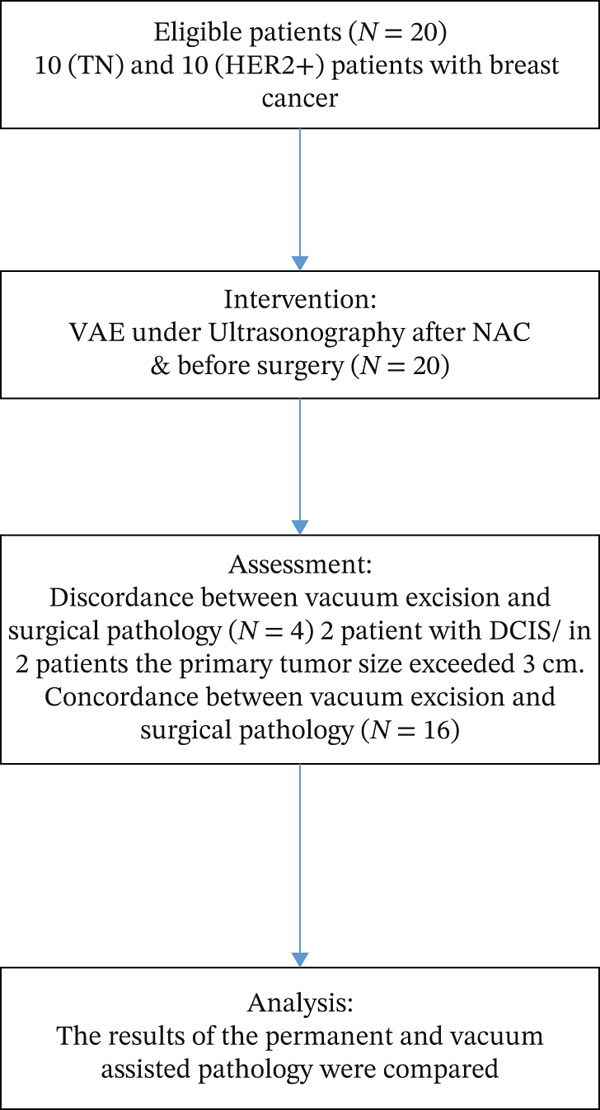
Study flow chart. Abbreviations: NAC, neoadjuvant chemotherapy; TN, triple negative; VAE, vacuum‐assisted excision.

Furthermore, the accuracy of vacuum excision in identifying residual tumors is 80%, indicating an 80% probability that patients will be correctly categorized based on the excision results (Figure 1).

## 4. Discussion

The findings of this study revealed an accuracy rate of 80% for the vacuum excision technique. Notably, the NPV of the vacuum excision approach was determined to be 76.5%, indicating that in 23.5% of cases (four patients) where residual tumor presence was confirmed, the vacuum excision results were falsely negative. Furthermore, among these cases, three instances were identified as DCIS, highlighting the need for careful selection of patients for vacuum excision as a possible alternative method for surgery.

In the multicenter cohort study by Loevezijn involving 167 post‐NAC patients undergoing vacuum excision, a notably higher false‐negative rate of 37% was observed in vacuum excision pathology reports, surpassing the findings of the current study [18].

NAC has emerged as the standard therapeutic approach for breast cancer, extending beyond advanced cases to encompass early‐stage and operable breast cancer cases. Notably, recent advancements in chemotherapy agents and targeted therapies have significantly elevated the pathologic complete response (PCR) rates in HER‐2 positive and TN breast cancer subtypes, reaching up to 60% [19, 20]. The consideration of mastectomy over breast‐conserving surgery has garnered attention in select breast cancer subtypes [21]. Globally, multiple prospective studies are underway to evaluate the safety and efficacy of mastectomy in patients exhibiting high response rates to NAC [21, 22]. Despite ongoing research efforts, existing prospective studies offer limited insights into the feasibility and outcomes associated with complete breast surgery, particularly in specific patient subgroups.

The higher NPV in their study may be due to the number of samples under investigation or to variations in the proficiency of the radiologists performing the vacuum excision. In a recent cohort study on 20 patients conducted by Rossi et al. in Italy, findings conferred a diagnostic accuracy rate of 90% for VAE in detecting residual tumor vs. surgical pathologic findings [23]. According to our findings, the two remaining patients were identified with DCIS. These observations suggest the potential utility of VAE as a way to eliminate the need for surgical intervention in these particular subsets of patients with breast cancer.

In the current study, a notable observation was made in four patients where the vacuum pathology report indicated the absence of residual tumor, yet surgical pathology revealed residual tumor presence, with three cases identified as DCIS. This discrepancy raises concerns regarding the challenge of completely eradicating non‐invasive foci, particularly DCIS, through NAC. The potential association of DCIS with IDC, coupled with the multifocality and dispersion characteristics of DCIS, may contribute to the incomplete clearance of tumor sites by the vacuum excision method. Surgical pathology reports, necessitating more extensive tissue removal, highlight the limitations of vacuum excision in comprehensively assessing tumor burden and guiding treatment decisions. This underscores the importance of cautious patient selection when considering vacuum excision as an alternative to surgery.

In a prospective investigation, Koelbel et al. assessed the diagnostic accuracy of vacuum‐assisted biopsy after NAC within a multicenter cohort derived from Elman‐based institutions. The cohort comprised 398 subjects who underwent a comparative procedure. The study’s outcomes elucidated a statistically significant association between the incidence of false‐negative results in vacuum‐assisted biopsy pathology and the presence of multicentric tumors, as well as the concurrence of invasive carcinoma with DCIS. Notably, an elevated rate of false‐negative findings was observed in instances where the radiological markers within the tumor mass were not effectively excised via vacuum‐assisted techniques. Upon excluding such cases, the false‐negative rate was recalculated at 2.9% among a subset of 104 patients. These findings suggest that vacuum‐assisted biopsy, when optimally implemented, constitutes a reliable modality for the excision of residual tumoral tissue following NAC [24].

In 2021, Khare et al. conducted a research study in India, focusing on a cohort of 18 patients with hormone receptor‐negative, HER2‐positive or HER2‐negative breast cancer, all of whom received NAC before surgical intervention. The cohort was subjected to vacuum‐assisted biopsies under ultrasonographic guidance performed by a radiologist in 10 of these patients. This interventional diagnostic technique was subsequently evaluated against surgical pathology outcomes to assess for the presence of residual tumor tissue. The study reported that vacuum‐assisted biopsy yielded a sensitivity of 50% in the detection of residual tumor, while specificity was recorded at 100%. When vacuum‐assisted biopsy was integrated with mammography and ultrasound, the combined modalities demonstrated an enhanced sensitivity of 77% and a specificity of 66% for discerning complete PCR. The findings underscored a concern regarding the limited sensitivity of vacuum‐assisted biopsy as a stand‐alone diagnostic tool in the accurate determination of residual tumor burden post‐neoadjuvant chemotherapy [25].

In a prospective study, Zhiqiang Shi et al. evaluated 132 patients with primary breast cancer who achieved either breast radiologic complete response (brCR) or partial response (brPR) following NAC. The investigators reported that ultrasound‐guided multiple‐site CNB was able to predict PCR after NAC with a high degree of accuracy. These findings provide a rationale for the selective omission of breast surgery in appropriately selected patients and may contribute to reducing postoperative morbidity, enhancing quality of life, and decreasing healthcare expenditures [26].

Moreover, the study findings revealed a statistical similarity between the results of vacuum excision pathology and surgical pathology in 16 patients, emphasizing the congruence between the two diagnostic modalities. While the significant *p* value of 0.004 underscores this consistency, achieving 100% diagnostic accuracy with vacuum excision remains imperative to avoid unnecessary surgeries and prevent potential disease recurrence. Even a single false negative pathology report from vacuum excision could have critical implications for patient outcomes, reinforcing the necessity for meticulous evaluation and patient selection.

In light of the study outcomes, it is evident that the vacuum excision method cannot presently replace surgery in patients with breast cancer. To validate these findings comprehensively, larger‐scale studies with rigorous patient selection criteria are warranted. Notably, recent research has explored the feasibility of surgical removal in specific breast cancer subgroups post‐confirmation of complete tumor response following NAC, demonstrating promising outcomes with extended patient follow‐up. However, patient consent and awareness of the risk of local recurrence are crucial considerations in such treatment approaches.

An additional consideration is the need for lymph node surgery on the affected side. Research in this area has been somewhat limited but has shown promising outcomes. In a study involving 258 patients with breast cancer post‐NAC, individuals with non‐involved lymph nodes pre‐NAC and the absence of residual tumor in breast vacuum biopsy may potentially skip lymph node surgery. However, further validation of this finding necessitates larger‐scale studies. Notably, our study did not assess axillary lymph nodes due to the technical expertise and experience required for vacuum excision in the axilla. This procedure is intricate and should ideally be performed in an operating room setting to ensure adequate resources for managing potential complications. The higher NPV in their study may be due to the sample size [27].

It is important to note that the evaluation of axillary lymph node status remains a crucial component in the overall assessment of patients with breast cancer. While vacuum biopsy demonstrates potential, continued advancements in technique and provider expertise are necessary to realize its clinical utility fully.

The current study is limited by a relatively small sample size, which may constrain the robustness and generalizability of the findings. Additionally, patient hesitancy towards undergoing vacuum excision procedures could impede the timely acquisition of adequate sample volumes, potentially compromising the accuracy of the analysis. Future research endeavors should prioritize studies with significantly larger participant cohorts to enhance the statistical power and representativeness of the results.

## 5. Conclusion

In conclusion, the findings of our study suggest that while vacuum excision holds promise as an alternative to surgical sampling for evaluating breast lesions, it cannot currently serve as a substitute for surgery in the management of patients with breast cancer. The accuracy of vacuum excision in identifying residual tumors was found to be 80%, indicating that it may not provide the level of precision required for comprehensive patient care. Further research with larger cohorts is warranted to expand our understanding in this area and provide more definitive guidance on the role of vacuum excision in the management of breast cancer.

## Author Contributions

**Shahpar Haghighat and Hawzhin Hashemi:** conceptualization, methodology, investigation, formal analyses, supervision, data curation, writing – original draft preparation, writing – review and editing. **Aseie Olfatbakhsh, Shiva Moghadam, Alireza Aghanajafi, Laila Haydari, Marzieh Rostami:** investigation, data collection, writing – original draft preparation, writing – review and editing.

## Funding

No funding was received for this manuscript.

## Disclosure

All of the authors read and approved the final draft of the manuscript.

## Ethics Statement

Ethical approval was obtained from the Clinical Research Ethics Committee of Motamed Cancer Institute. Written informed consent was obtained from each patient.

## Conflicts of Interest

The authors declare no conflicts of interest.

## Data Availability

The data that support the findings of this study are available on request from the corresponding author. The data are not publicly available due to privacy or ethical restrictions.

## References

[1] Fisher B. , Bryant J. , Wolmark N. , Mamounas E. , Brown A. , Fisher E. R. , Wickerham D. L. , Begovic M. , DeCillis A. , Robidoux A. , Margolese R. G. , Cruz A. B. , Hoehn J. L. , Lees A. W. , Dimitrov N. V. , and Bear H. D. , Effect of Preoperative Chemotherapy on the Outcome of Women With Operable Breast Cancer, Journal of Clinical Oncology. (1998) 16, no. 8, 2672–2685, 10.1200/jco.1998.16.8.2672, 2-s2.0-0031926839. 9704717

[2] Rastogi P. , Anderson S. J. , Bear H. D. , Geyer C. E. , Kahlenberg M. S. , Robidoux A. , Margolese R. G. , Hoehn J. L. , Vogel V. G. , Dakhil S. R. , Tamkus D. , King K. M. , Pajon E. R. , Wright M. J. , Robert J. , Paik S. , Mamounas E. P. , and Wolmark N. , Preoperative Chemotherapy: Updates of National Surgical Adjuvant Breast and Bowel Project Protocols B-18 and B-27, Journal of Clinical Oncology. (2008) 26, no. 5, 778–785, 10.1200/JCO.2007.15.0235, 2-s2.0-39149111633, 18258986. 18258986

[3] Cheng X. , Li Y. , Liu B. , Xu Z. , Bao L. , and Wang J. , 18F-FDG PET/CT and PET for Evaluation of Pathological Response to Neoadjuvant Chemotherapy in Breast Cancer: A Metaanalysis, Acta Radiologica. (2012) 53, no. 6, 615–627, 10.1258/ar.2012.110603, 2-s2.0-84864031130, 22734080. 22734080

[4] Marinovich M. L. , Houssami N. , Macaskill P. , Michael L. , Sardanelli F. , Irwig L. , Mamounas E. P. , Minckwitz G. , Brennan M. E. , and Ciatto S. , Meta-Analysis of Magnetic Resonance Imaging in Detecting Residual Breast Cancer After Neoadjuvant Therapy, Journal of the National Cancer Institute. (2013) 105, no. 5, 321–333, 10.1093/jnci/djs528, 2-s2.0-84874855647, 23297042. 23297042

[5] Montagna E. , Bagnardi V. , Rotmensz N. , Viale G. , Pruneri G. , Veronesi P. , Cancello G. , Balduzzi A. , Dellapasqua S. , Cardillo A. , Luini A. , Zurrida S. , Gentilini O. , Mastropasqua M. G. , Bottiglieri L. , Iorfida M. , Goldhirsch A. , and Colleoni M. , Pathological Complete Response After Preoperative Systemic Therapy and Outcome: Relevance of Clinical and Biologic Baseline Features, Breast Cancer Research and Treatment. (2010) 124, no. 3, 689–699, 10.1007/s10549-010-1027-4, 2-s2.0-78649330819, 20625816. 20625816

[6] Weiss A. , Bashour S. I. , Hess K. , Thompson A. M. , and Ibrahim N. K. , Effect of Neoadjuvant Chemotherapy Regimen on Relapse-Free Survival Among Patients With Breast Cancer Achieving a Pathologic Complete Response: An Early Step in the De-Escalation of Neoadjuvant Chemotherapy, Breast Cancer Research. (2018) 20, no. 1, 10.1186/s13058-018-0945-7, 2-s2.0-85045413901, 29661243. PMC5902970 29661243

[7] Yan la Parra R. F. and Kuerer H. M. , Selective Elimination of Breast Cancer Surgery in Exceptional Responders: Historical Perspective and Current Trials, Breast Cancer Research. (2016) 18, no. 1, 10.1186/s13058-016-0684-6, 2-s2.0-85008628458, 26951131. PMC4782355 26951131

[8] Gianni L. , Pienkowski T. , Im Y. H. , Roman L. , Tseng L. M. , Liu M. C. , Lluch A. , Staroslawska E. , Haba-Rodriguez J. , Im S. , Pedrini J. L. , Poirier B. , Morandi P. , Semiglazov V. , Srimuninnimit V. , Bianchi G. , Szado T. , Ratnayake J. , Ross G. , and Valagussa P. , Efficacy and Safety of Neoadjuvant Pertuzumab and Trastuzumab in Women With Locally Advanced, Inflammatory, or Early HER2-Positive Breast Cancer (NeoSphere): A Randomised Multicentre, Open-Label, Phase 2 Trial, Lancet Oncology. (2012) 13, no. 1, 25–32, 10.1016/S1470-2045(11)70336-9, 2-s2.0-84855297353, 22153890. 22153890

[9] Dominici L. S. , Negron Gonzalez V. M. , Buzdar A. U. , Lucci A. , Mittendorf E. A. , Le-Petross H. , Babiera G. V. , Meric-Bernstam F. , Hunt K. K. , and Kuerer H. M. , Cytologically Proven Axillary Lymph Node Metastases are Eradicated in Patients Receiving Preoperative Chemotherapy With Concurrent Trastuzumab for HER2-Positive Breast Cancer, Cancer. (2010) 116, no. 12, 2884–2889, 10.1002/cncr.25152, 2-s2.0-77954021905, 20564395. 20564395 PMC4361091

[10] De Los Santos J. F. , Cantor A. , Amos K. D. , Forero A. , Golshan M. , Horton J. K. , Hudis C. A. , Hylton N. M. , McGuire K. , Meric-Bernstam F. , Meszoely I. M. , Nanda R. , and Hwang E. S. , Magnetic Resonance Imaging as a Predictor of Pathologic Response in Patients Treated With Neoadjuvant Systemic Treatment for Operable Breast Cancer. Translational Breast Cancer Research Consortium trial 017, Cancer. (2013) 119, no. 10, 1776–1783, 10.1002/cncr.27995, 2-s2.0-84877586179, 23436342. 23436342 PMC3939707

[11] Peintinger F. , Kuerer H. M. , Anderson K. , Boughey J. C. , Meric-Bernstam F. , Singletary S. E. , Hunt K. K. , Whitman G. J. , Stephens T. , Buzdar M. U. , Green M. C. , and Symmans W. F. , Accuracy of the Combination of Mammography and Sonography in Predicting Tumor Response in Breast Cancer Patients After Neoadjuvant Chemotherapy, Annals of Surgical Oncology. (2006) 13, no. 11, 1443–1449, 10.1245/s10434-006-9086-9, 2-s2.0-33750986214, 17028770. 17028770

[12] Schaefgen B. , Mati M. , Sinn H. P. , Golatta M. , Stieber A. , Rauch G. , Hennigs A. , Richter H. , Domschke C. , Schuetz F. , Sohn C. , Schneeweiss A. , and Heil J. , Can Routine Imaging After Neoadjuvant Chemotherapy in Breast Cancer Predict Pathologic Complete Response?, Annals of Surgical Oncology. (2016) 23, no. 3, 789–795, 10.1245/s10434-015-4918-0, 2-s2.0-84957846457, 26467456. 26467456

[13] Hayashi N. , Takahashi Y. , Matsuda N. , Tsunoda H. , Yoshida A. , Suzuki K. , Nakamura S. , and Yamauchi H. , The Prognostic Effect of Changes in Tumor Stage and Nodal Status After Neoadjuvant Chemotherapy in Each Primary Breast Cancer Subtype, Clinical Breast Cancer. (2018) 18, no. 2, e219–e229, 10.1016/j.clbc.2017.09.013, 2-s2.0-85033585785, 29138067. 29138067

[14] Richter H. , Hennigs A. , Schaefgen B. , Markus Hahn M. , Blohmer J. U. , Kümmel S. , Kühn T. , Thill M. , Friedrichs K. , Sohn C. , Golatta M. , and Heil J. , Is Breast Surgery Necessary for Breast Carcinoma in Complete Remission Following Neoadjuvant Chemotherapy?, Geburtshilfe Frauenheilkd. (2018) 78, no. 1, 48–53, 10.1055/s-0043-124082, 2-s2.0-85055772335, 29375145. 29375145 PMC5778196

[15] Kuerer H. M. , Rauch G. M. , Krishnamurthy S. , Adrada B. E. , Caudle A. S. , DeSnyder S. M. , Black D. M. , Santiago L. , Hobbs B. P. , Lucci A. , Gilcrease M. , Hwang R. F. , Candelaria R. P. , Chavez-Mac Gregor M. , Smith B. D. , Arribas E. , Moseley T. , Teshome M. , Miggins M. V. , Valero V. , Hunt K. K. , and Yang W. T. , A Clinical Feasibility Trial for Identification of Exceptional Responders in Whom Breast Cancer Surgery Can Be Eliminated Following Neoadjuvant Systemic Therapy, Annals of Surgery. (2018) 267, no. 5, 946–951, 10.1097/SLA.0000000000002313, 2-s2.0-85019718572, 28549010. 28549010 PMC6051523

[16] Kuerer H. M. , Smith B. D. , Krishnamurthy S. , Yang W. T. , Valero V. , Shen Y. , Lin H. , Lucci A. , Boughey J. C. , White R. L. , Diego E. J. , Rauch G. M. , Moseley T. W. , van la Parra R. F. D. , Adrada B. E. , Leung J. W. T. , Sun S. X. , Teshome M. , Miggins M. V. , Hunt K. K. , DeSnyder S. M. , Ehlers R. A. , Hwang R. F. , Colen J. S. , Arribas E. , Samiian L. , Lesnikoski B. A. , Piotrowski M. , Bedrosian I. , Chong C. , Refinetti A. P. , Huang M. , Candelaria R. P. , Loveland-Jones C. , Mitchell M. P. , and Shaitelman S. F. , Eliminating Breast Surgery for Invasive Breast Cancer in Exceptional Responders to Neoadjuvant Systemic Therapy: A Multicentre, Single-Arm, Phase 2 Trial, Lancet Oncology. (2022) 23, no. 12, 1517–1524, 10.1016/S1470-2045(22)00613-1, 36306810. 36306810

[17] Haque W. , Verma V. , Hatch S. , Suzanne Klimberg V. , Klimberg V. S. , Butler E. B. , and Teh B. S. , Response Rates and Pathologic Complete Response by Breast Cancer Molecular Subtype Following Neoadjuvant Chemotherapy, Breast Cancer Research and Treatment. (2018) 170, no. 3, 559–567, 10.1007/s10549-018-4801-3, 2-s2.0-85045875481. 29693228

[18] van Loevezijn A. A. , van der Noordaa M. E. M. , van Werkhoven E. D. , Loo C. E. , Winter-Warnars G. A. O. , Wiersma T. , van de Vijver K. K. , Groen E. J. , Blanken-Peeters C. F. J. M. , Zonneveld B. J. G. L. , Sonke G. S. , van Duijnhoven F. H. , and Vrancken Peeters M. T. F. D. , Minimally Invasive Complete Response Assessment of the Breast After Neoadjuvant Systemic Therapy for Early Breast Cancer (MICRA Trial): Interim Analysis of a Multicenter Observational Cohort Study, Annals of Surgical Oncology. (2021) 28, no. 6, 3243–3253, 10.1245/s10434-020-09273-0, 33263830. 33263830 PMC8119397

[19] Loibl S. , Jackisch C. , Schneeweiss A. , Schmatloch S. , Aktas B. , Denkert C. , Wiebringhaus H. , Kümmel S. , Warm M. , Paepke S. , Just M. , Hanusch C. , Hackmann J. , Blohmer J. U. , Clemens M. , Dan Costa S. , Gerber B. , Engels K. , Nekljudova V. , von Minckwitz G. , Untch M. , and investigators of the German Breast Group (GBG) and the Arbeitsgemeinschaft Gynäkologische Onkologie—Breast (AGO-B) study groups , Dual HER2-Blockade With Pertuzumab and Trastuzumab in HER2-Positive Early Breast Cancer: A Subanalysis of Data From The Randomized Phase III GeparSepto Trial, Annals of Oncology. (2017) 28, no. 3, 497–504, 10.1093/annonc/mdw610, 2-s2.0-85018306603, 27831502. 27831502

[20] Schmid P. , Cortes J. , Pusztai L. , McArthur H. , Kümmel S. , Bergh J. , Denkert C. , Park Y. H. , Hui R. , Harbeck N. , Takahashi M. , Foukakis T. , Fasching P. A. , Cardoso F. , Untch M. , Jia L. , Karantza V. , Zhao J. , Aktan G. , Dent R. , and O′Shaughnessy J. , Pembrolizumab for Early Triple-Negative Breast Cancer, New England Journal of Medicine. (2020) 382, no. 9, 810–821, 10.1056/NEJMoa1910549. 32101663

[21] Gharzai L. A. , Szczygiel L. A. , Shumway D. A. , Bandos H. , Julian T. B. , Mamounas E. P. , White J. , De Los Santos J. F. , Basik M. , Ganz P. A. , and Jagsi R. , A Qualitative Study to Evaluate Physician Attitudes Regarding Omission of Surgery Among Exceptional Responders to Neoadjuvant Systemic Therapy for Breast Cancer (NRG-CC006), Breast Cancer Research and Treatment. (2021) 187, no. 3, 777–784, 10.1007/s10549-021-06172-0, 33740205. 33740205 PMC8648089

[22] Shigematsu H. , Fujisawa T. , Shien T. , and Iwata H. , Omitting Surgery for Early Breast Cancer Showing Clinical Complete Response to Primary Systemic Therapy, Japanese Journal of Clinical Oncology. (2020) 50, no. 6, 629–634, 10.1093/jjco/hyaa055, 32378709. 32378709

[23] Rossi E. M. C. , Invento A. , Pesapane F. , Pagan E. , Bagnardi V. , Fusco N. , Venetis K. , Dominelli V. , Trentin C. , Cassano E. , Gilardi L. , Mazza M. , Lazzeroni M. , De Lorenzi F. , Caldarella P. , De Scalzi A. , Girardi A. , Sangalli C. , Alberti L. , Sacchini V. , Galimberti V. , and Veronesi P. , Diagnostic Performance of Image-Guided Vacuum-Assisted Breast Biopsy After Neoadjuvant Therapy for Breast Cancer: Prospective Pilot Study, British Journal of Surgery. (2023) 110, no. 2, 217–224, 10.1093/bjs/znac391, 36477768. 36477768 PMC10364486

[24] Koelbel V. , Pfob A. , Schaefgen B. , Sinn P. , Feisst M. , Golatta M. , Gomez C. , Stieber A. , Bach P. , Rauch G. , and Heil J. , Vacuum-Assisted Breast Biopsy After Neoadjuvant Systemic Treatment for Reliable Exclusion of Residual Cancer in Breast Cancer Patients, Annals of Surgical Oncology. (2022) 29, no. 2, 1076–1084, 10.1245/s10434-021-10847-9, 34581923. 34581923 PMC8724060

[25] Khare S. , Santosh I. , Laroiya I. , Singh T. , Bal A. , and Singh G. , Assessment of Pathological Complete Response Using Vacuum-Assisted Biopsy in Breast Cancer Patients Who Have Clinical and Radiological Complete Response After Neo-Adjuvant Chemotherapy, Basic and Clinical Research. (2023) 17, 1–7, 10.1177/11782234231205698, 38024141. PMC10655653 38024141

[26] Shi Z. , Qiu P. , Wang Y. , and Liu H. , Minimally Invasive Biopsy Technique Predicting Breast Pathological Complete Response After Neoadjuvant Therapy for Breast Cancer, Gland Surgery. (2025) 14, no. 7, 1263–1271, 10.21037/gs-2025-103, 40771373. 40771373 PMC12322751

[27] Chen R. , Li S. , Li Y. , Zhu Q. , Shi X. , Xu L. , Xu Y. , Zhang W. , Huang X. , Wang J. , and Zha X. , Can Axillary Surgery Be Omitted in Patients With Breast Pathologic Complete Response After Neoadjuvant Systemic Therapy for Breast Cancer? A Real-World Retrospective Study in China, Journal of Cancer Research and Clinical Oncology. (2021) 147, no. 12, 3495–3501, 10.1007/s00432-021-03763-8, 34398298. 34398298 PMC11802028

